# Bilateral Choroidal Osteoma in a Teenage Girl With Chronic Posterior Scleritis

**DOI:** 10.7759/cureus.27136

**Published:** 2022-07-22

**Authors:** Emily Ming Choo Ng, Amelia Lay Suan Lim, Sangeeta Kuganasan, Hanizasurana Hashim

**Affiliations:** 1 Ophthalmology, University Kebangsaan Malaysia, Kuala Lumpur, MYS; 2 Ophthalmology, Hospital Selayang, Selangor, MYS

**Keywords:** igg4-related disease, tumour growth, posterior scleritis, multimodal imaging, choroidal osteoma

## Abstract

A 14-year-old girl with bilateral chronic posterior scleritis was referred to us for poor control of ocular inflammation. There was an incidental finding of choroidal osteoma bilaterally whereby the choroidal mass in her right eye demonstrated a significant tumor growth in a short duration of two months. The right eye choroidal tumor was orangy in color with a well-defined margin, two-disc diameter in size, and located at the macula encroaching the fovea. Multimodal imaging assessments, including serial color fundus photo, enhanced depth imaging optical coherence tomography (EDI-OCT), and B scan ultrasonography monitoring, confirmed a continuous rapid growth of choroidal osteoma with episodes of scleritis flare-ups. Furthermore, intolerance toward second-line immunosuppressants and loss of follow-ups during the coronavirus disease 2019 (COVID-19) pandemic lockdown led to frequent relapses of her posterior scleritis. Therefore, a new treatment plan was designed, and close monitoring of choroidal osteoma growth and control of posterior scleritis were initiated. Subsequently, bilateral posterior scleritis remained quiescent, and her vision remained stable with stagnant growth of choroidal osteoma.

## Introduction

Choroidal osteoma is a rare, benign osseous tumor within the choroid. It is usually unilateral and occurs in the juxtapupillary region of the eyes in healthy women in the second to third decades of life. Choroidal osteomas tend to be orange-red in color in the early stages, and later develop a yellowish tint secondary to retinal pigment epithelium (RPE) depigmentation [1]. It was first reported by Gass et al. in 1978 [2]. They documented the cases of four young females with choroidal osteoma, and no risk factors were identified. We report a case of bilateral chronic posterior scleritis with secondary choroidal osteoma.

## Case presentation

A 14-year-old girl with a prior history of bilateral posterior scleritis treated at a secondary hospital was referred to a facility in the central region of Malaysia for resistant steroid therapy for scleritis control. She underwent a thorough autoimmune and infective workup, but the results were unremarkable. Initially, she was started on oral methotrexate (MTX) 15 mg per week combined with gradual tapering of oral prednisolone. However, she developed flare-ups when oral prednisolone was tapered to 10 mg OD or below. Subsequently, she was co-managed with the Pediatric Rheumatology team for the optimization of second-line treatment. Oral MTX was switched to the subcutaneous route, given poor tolerance to the oral form. After that, scleritis control was achieved and maintained for about a year. However, after a year of being quiescent, she developed frequent relapses in her right eye. Hence, subcutaneous MTX was further increased to 17.5 mg per week, and oral prednisolone was escalated to an immunosuppressive dose. These improved the flare-ups, but upon tapering of prednisolone below 20 mg, she began to experience mild aches over her right eye. She was subsequently referred to the Uveitis Clinic by the Pediatric Rheumatology team.

**Figure 1:**
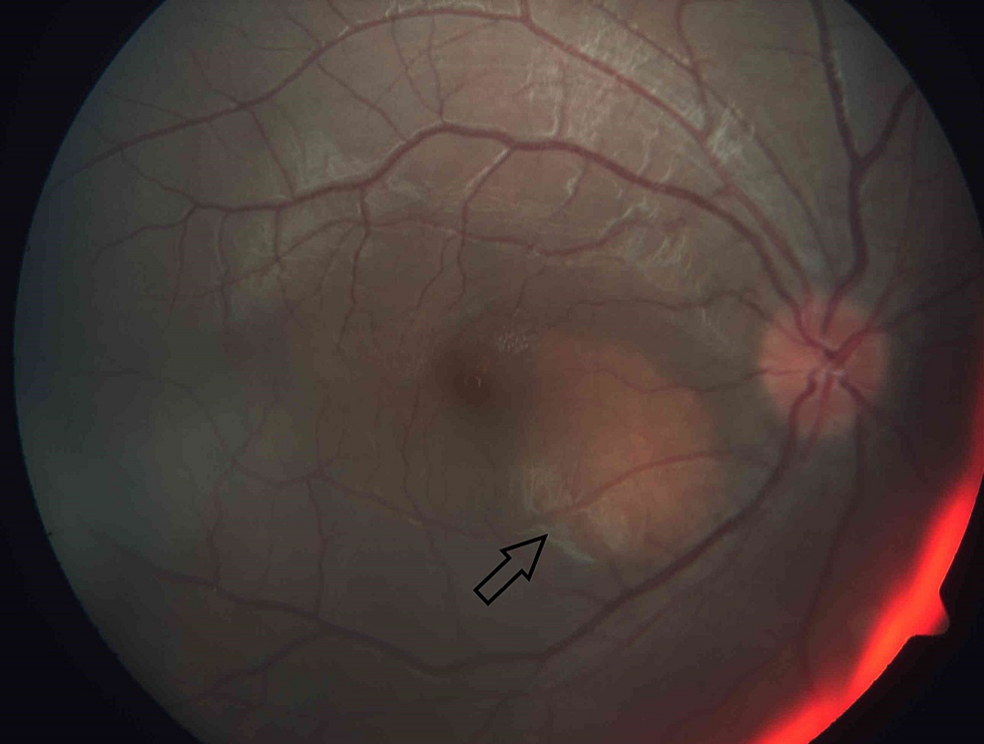
An orangy choroidal mass (arrow) measuring 2-disc diameter located at the macula region in the right eye

**Figure 2:**
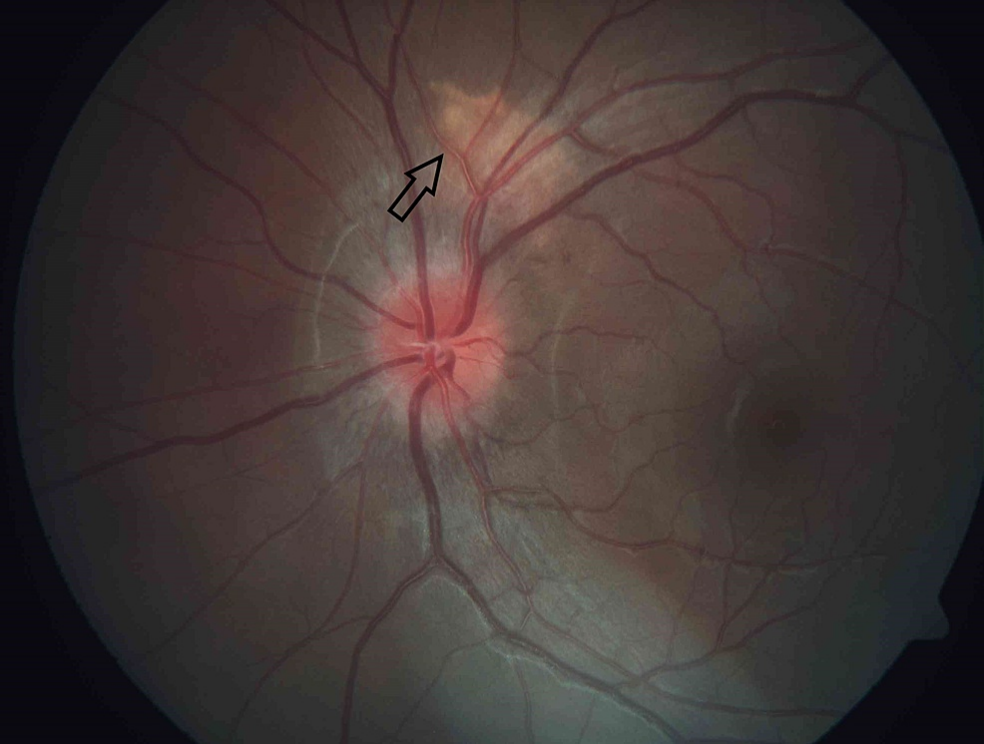
An orangy choroidal mass (arrow) measuring 1-disc diameter located at the superior juxtapapillary area in the left eye

Upon our first review, her visual acuity for both eyes was 20/30. Anterior segments were unremarkable, and intraocular pressures were normal. There were no signs of intraocular inflammation or steroid toxicity. However, we noted bilateral optic disc hyperemia. There was an incidental finding of a two-disc diameter orangy choroidal mass at the macular region in her right eye (Figure 1) and a one-disc diameter orangy choroidal mass at the superior juxtapapillary area in her left eye (Figure 2).

**Figure 3:**
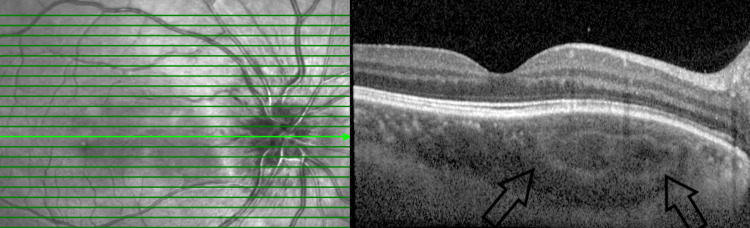
Right eye choroidal mass with sponge-like pattern with multilayer configuration and a visible sclero-choroidal junction. The inner and outer retina are intact, and no subretinal fluid is seen

**Figure 4:**
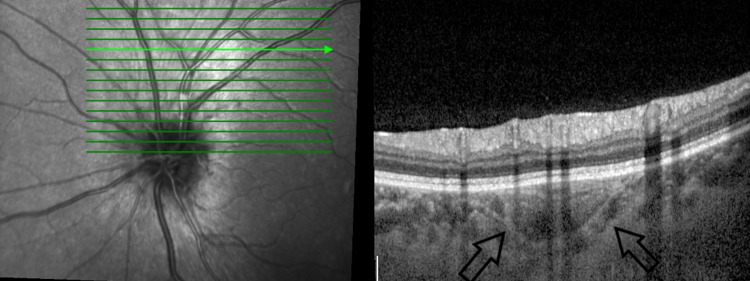
Left eye choroidal mass with a similar sponge-like pattern with multilayer configuration and a visible sclero-choroidal junction

**Figure 5:**
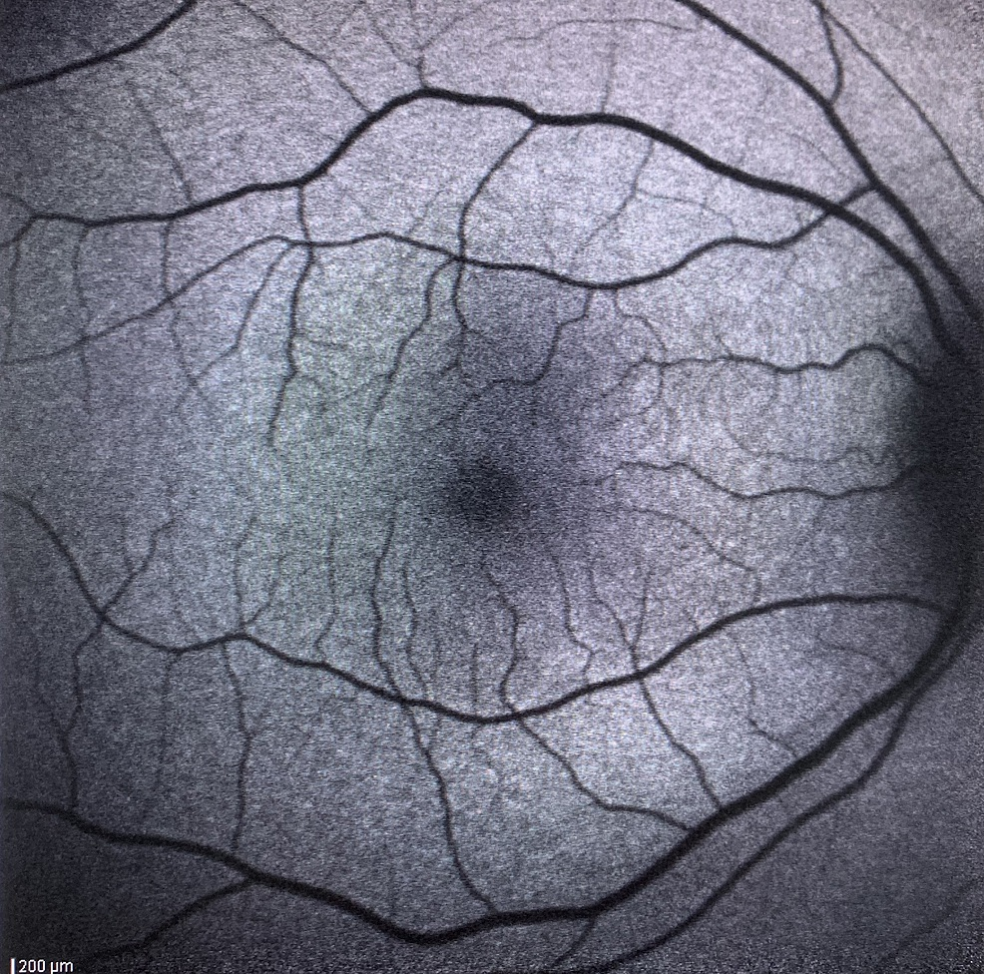
Right eye with relative normal fundus autofluorescence

**Figure 6:**
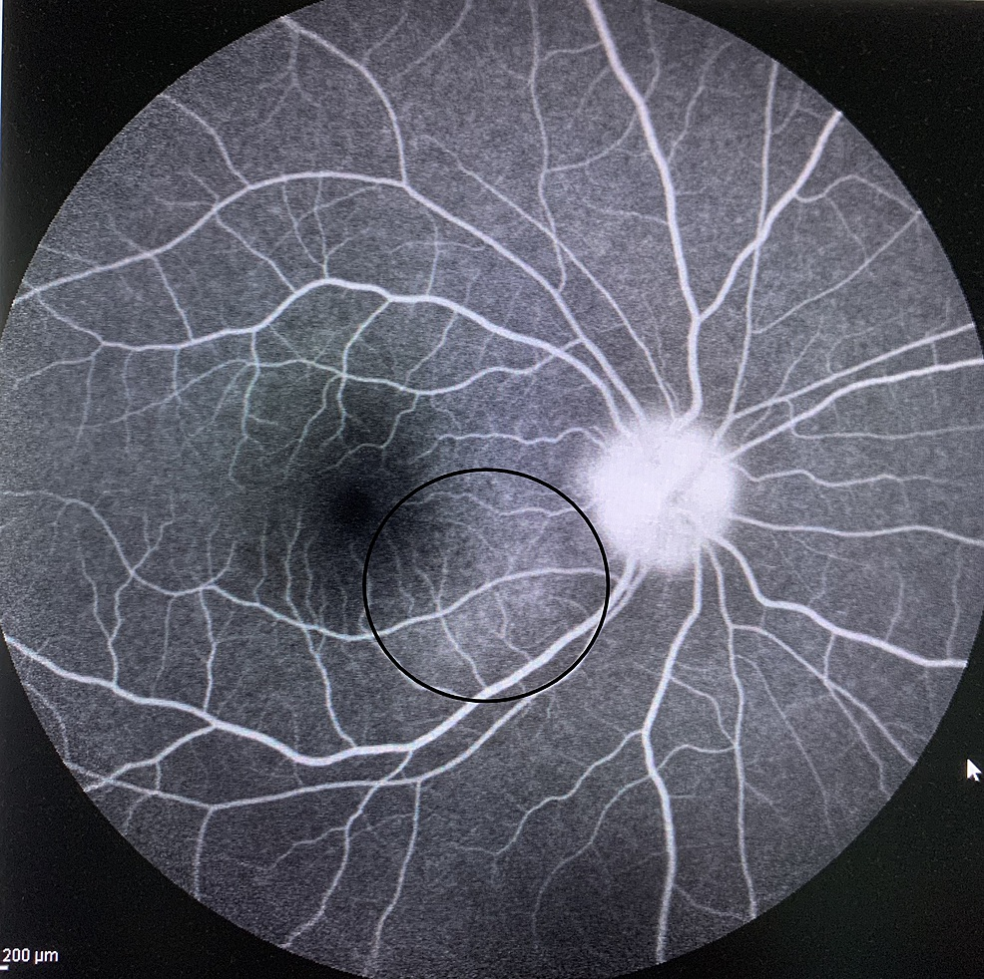
Right eye choroidal lesion with late hyperfluorescence staining (circle) on fluorescence angiography

**Figure 7:**
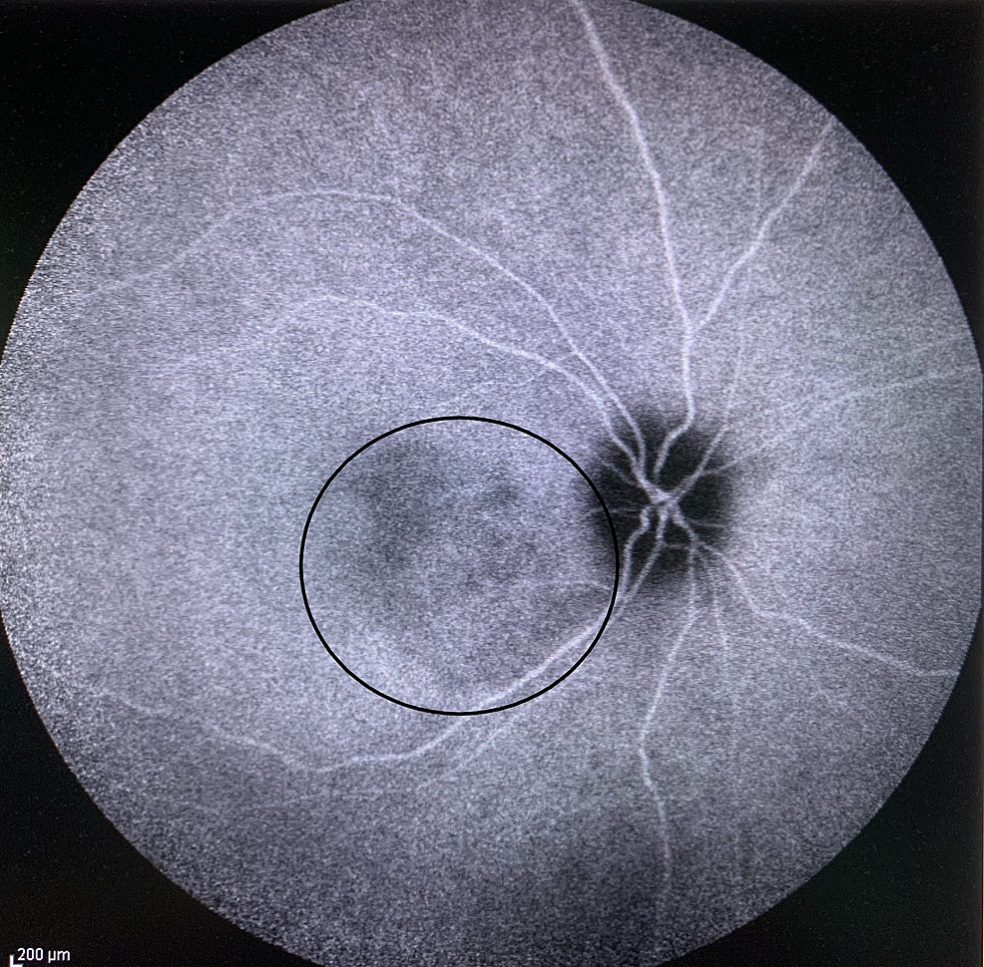
Right eye choroidal lesion with hypocyanescence (circle) on indocyanine-green angiography

Enhanced depth imaging optical coherence tomography (EDI-OCT) demonstrated a mass with a spongy form appearance in the choroidal layer (Figures 3-4) [3]. There was no subretinal or intraretinal fluid seen. On the B scan, the posterior scleral thickness was 1.8 mm for the right eye and 1.5 mm for the left eye. T-sign was absent in both eyes. In addition, a B scan on both eyes revealed highly reflective choroidal lesions with posterior acoustic shadowing consistent with bone density, suggesting dense calcifications. On fundus autofluorescence (FAF), there were no abnormalities detected in both eyes as the overlying RPE layers were intact (Figure 5). On fluorescein angiography (FFA), the optic disc hyperfluorescent and the choroidal lesions showed early hypofluorescence and later hyperfluorescence (Figure 6). There was no choroidal neovascularization as there was an absence of leakage on FFA. On indocyanine green angiography (ICG), bilateral choroidal lesions showed hypocyanescence (Figure 7).

**Figure 8:**
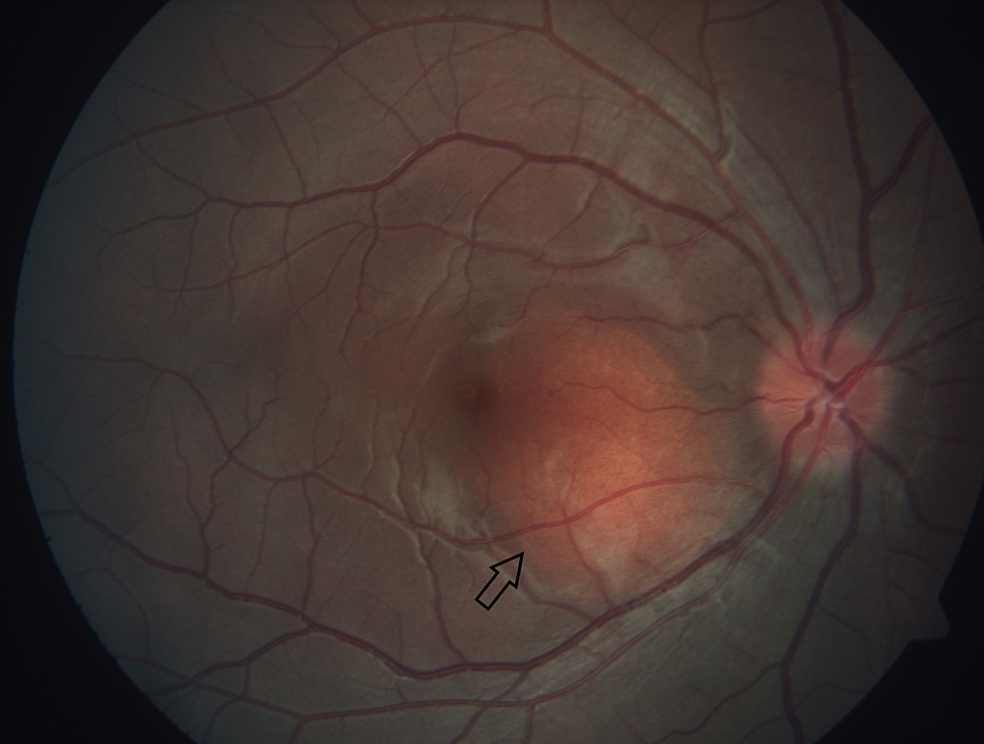
Right eye choroidal mass has increased in size to 3-disc diameter, encroaching the fovea

Uveitic workup was repeated. The Mantoux test, TB Quantiferon, anti-nuclear antibodies (ANA), rheumatoid factor (RF), antineutrophil cytoplasmic antibodies (ANCA), and venereal disease research laboratories (VDRL) were all negative. Therefore, she was treated as a case of bilateral choroidal osteoma likely induced by poorly controlled posterior scleritis. She was continued on oral prednisolone 15 mg OD and subcutaneous MTX 17.5 mg per week. After two months, the right eye choroidal mass significantly increased in size, encroaching the fovea (Figure 8), while the left eye choroidal lesion remained unchanged. A decision was made to switch MTX to oral CellCept (mycophenolate mofetil) due to poor pain control with MTX. Her disease remained stable with oral CellCept 1 gm in the morning and 750 mg at night. The oral prednisolone dose was tapered down to 10 mg OD, without further flare-ups. Due to recurrent scleritis, IgG4 blood was taken, and it was found to be within the normal range of 0.049-1.985 (0.526 g/L).

## Discussion

Although the characteristic clinical appearance of choroidal osteoma has been extensively described in the literature [4-6], due to its rarity, Browning [7] found that approximately 90% of eye care providers missed its diagnosis at initial presentations.

Despite the availability of well-documented case reports and reviews [2,4,8], the etiology and pathogenesis of choroidal osteoma are still not well established. However, it has been linked to intraocular inflammation [9-10], pregnancy [11], trauma [12], hereditary causes [13], and osseous choristoma [14]. In our case, chronic posterior scleritis was the precipitating cause of the enlargement of the choroidal osteoma.

On histopathology, choroidal osteoma consists of bony trabeculae between an altered choriocapillaris and the outer choroidal layers [15]. In addition, it comprises osteoblasts, osteocytes, and osteoclasts, which suggest the presence of active bony remodeling. Thus, the pathogenesis of the growth of the choroidal osteoma, in this case, was likely secondary to inflammation. These explained the increasing size of the choroidal osteoma of the right eye when the patient had poorly controlled posterior scleritis. Subsequently, the posterior scleritis remained quiescent with the switching of the second-line immunosuppressant, and serial follow-ups revealed stagnant growth of the choroidal mass. Her visual acuity remained 20/30 in both eyes at the time of writing this report.

Subretinal mass in posterior scleritis is not uncommon [16-18]. Nodular posterior scleritis can present in a similar fashion to a choroidal mass with high internal reflectivity on B scan ultrasonography [16]. However, posterior acoustic shadowing giving a pseudo-optic disc appearance is highly suggestive of choroidal osteoma. FFA, ICG, and EDI-OCT findings in our patient were consistent with the findings in the literature.

Concurrent choroidal osteoma and posterior scleritis are rare. Based on our literature review, this is likely the third case report to describe it. The previous two case reports were published by Trimble et al. [9] and Nair et al. [19]. Though uncommon, the possibility of concurrent choroidal osteoma in a patient with posterior scleritis should not be ruled out.

We are also still considering the possibility of IgG4-related inflammatory disease due to the recurrent "behavior" of posterior scleritis and choroidal osteoma association in our patient, based on the study by Nair et al. [19]. However, the IgG4 level was found to be normal, which could be due to the disease being under control when the blood was taken.

## Conclusions

Choroidal osteoma is a rare benign ossifying tumor of the choroid. This case report highlights the enlargement of the choroidal osteoma with recurrent posterior scleritis, and clinicians should be vigilant about its presence. Decalcification of the tumor, choroidal neovascularization, and RPE atrophy can result in potential blindness. Hence, early detection, good control of ocular inflammation, and continuous monitoring of the tumor growth with multimodal imaging are essential to prevent permanent blindness in these patients.
